# Quantifying interaction uncertainty between subwatersheds and base-flow partitions on hydrological processes

**DOI:** 10.1371/journal.pone.0261859

**Published:** 2022-03-01

**Authors:** Bing Yan, Yi Xu

**Affiliations:** 1 Hydrology and Water Resources Department, Nanjing Hydraulic Research Institute, Nanjing, China; 2 College of Hydrology and Water Resources, Hohai University, Nanjing, China; 3 State Key Laboratory of Hydrology Water Resources and Hydraulic Engineering, Nanjing, China; TDTU: Ton Duc Thang University, VIET NAM

## Abstract

Base flow, as an important component of runoff, is the main recharge source of runoff during the dry period, especially in the Yellow River Basin located in a semiarid area. However, the process of obtaining base flow has great uncertainty when considering hydrological simulations. Thus, in this study, a three-step framework is proposed, i.e., the particle swarm optimization (PSO) algorithm is used to calibrate model parameters under different subbasin partitioning schemes; then, the hydrograph separation (HYSEP), Improved United Kingdom Institute of Hydrology (IUKIH) and Lyne and Hollick filter (Lyne-Hollick) methods are used to separate the baseflow from the total runoff process, thereby exploring the uncertainty impacts of baseflow segmentation methods on the hydrological simulation process. The subsample-variance-decomposition method is used to quantify the independent and interactive uncertainty in the hydrological simulation process. The results show that the Topmodel model can be better applied to the source area of the Yellow River (the *KGE* values in the Sub5, Sub13, Sub21, Sub29, Sub37 and Sub13 scenarios were 0.91 and 0.65, 0.94 and 0.86, 0.94 and 0.88, 0.92 and 0.82, 0.95 and 0.89, and 0.92 and 0.83, respectively). The subbasin division uncertainty had less impact on simulated streamflow during the dry season and had a significant impact in the wet season, such as, the subbasin division uncertainty caused the difference between the median of the simulated streamflow to be as high as 213.09 m^3^/s in August but only 107.19 m^3^/s in January; Meanwhile, the baseflow segmentation method uncertainty has a significant impact on the annual mean streamflow values under different subbasin segmentation schemes. In addition, the baseflow values estimated by the Lyne-Hollick and HYSEP methods were obviously higher than those estimated by the IUKIH method during the wet season. The uncertainty influence of subbasin partitioning schemes and baseflow segmentation methods had significant differences on hydrological processes in different periods. The uncertainty influence of subbasin partitioning schemes was dominant in the dry season, accounting for 86%, and the baseflow segmentation methods took second place, accounting for approximately 12%. In the wet season, the uncertainty influence of the baseflow segmentation methods was gradually weakened, which may have been due to the uncertainty influence of the hydrological model. These results provide a reference for the calibration and validation of hydrological model parameters using baseflow components.

## 1 Introduction

Climate change and high-intensity human activities accelerate the hydrological cycle. Hydrological models are an important tool to simulate hydrological processes and predict changes in water resources [1, 2]. In addition, distributed, semidistributed or lumped units indicates a critical way to differentiate hydrological models, as this is closely related to the scale at which the input variables are considered homogeneous. For the hydrological model, the distributed hydrological model response unit is a regular cell grid, the semidistributed hydrological model response unit is a subbasin, and the basin as a whole is evaluated as lumped. Watershed partitioning is widely used in semidistributed hydrological models to consider spatial heterogeneities in different areas within watersheds. However, many studies have been conducted by investigating the influence of watershed partitioning schemes on the model results of different hydrological models [3–5]. Several studies have discussed the influence of watershed partitioning schemes on the accuracy of hydrological simulations [6, 7]. Arabi et al. found that the accuracy of runoff simulation increased with increasing subbasin partitioning, but there was a threshold effect, which led to the stability or decline of runoff simulation accuracy [8]. Han et al. analyzed the influence of eight watershed partitioning schemes on SLURP hydrological simulations in the Xiangxi River Basin [9]. Jha et al. used the Soil and Water Assessment Tool (SWAT) model to determine the appropriate level of watershed partitioning for simulating flow, sediment, and nutrients over 30 years in four Iowa watersheds [10].

Recently, considerable attention has been focused on the uncertainties in hydrological models [11–13], such as parameter uncertainty, structural uncertainty, and input/output uncertainty. Many uncertainty assessment frameworks have been developed in the literature [14–16]. Research results under these assessment frameworks revealed that exploring the effects of these uncertainties on hydrological models has great significance for understanding hydrological processes. Moreover, what needs special attention is the uncertainty of the hydrological model parameters; hydrologists usually define a unique set of values as the calibration parameters for the model, whereas this is a difficult task because they need to address the equifinality issue. This indicates that there might be multiple acceptable sets of parameters that can represent hydrological processes of the basin [14]. Beven and Binley also showed that identical model results could be obtained using different parameter combinations, which indicates that similar streamflows can be modeled with different combinations of surface runoff and baseflow [14, 16–18]. At present, the uncertainty related research mainly focuses on the analysis of precipitation input, model parameter error and other factors, while there are few cases to explore the impact of data error on the uncertainty analysis of hydrological forecast and simulation results from the perspective of model verification basis. Flow series has become the most common basis for hydrological model validation because of its good characteristics of long series continuous monitoring and acquisition. However, flow series is one of the important factors affecting the simulation effect of hydrological model. It is very important to select appropriate and reliable flow series as the basis for model validation. Although at present, most theories assume that the measured flow data is true and effective and can be directly used for model calibration in hydrological simulation, according to relevant studies, the flow data may have large errors and uncertainties due to the heterogeneity of hydrological elements and monitoring level. Base flow, as an important component of runoff, is the main recharge source of runoff during the dry period, especially in the Yellow River Basin in semiarid and semihumid climate areas. In the dry season, the base flow accounts for more than 65.20% of the peak flow at the outlet section of the basin. At this time, the existence of base flow may have a far-reaching impact on the use of flow series calibration model, which can not be ignored. In the past, the application of base flow data for hydrological model verification has achieved better results. Such as, Rouhani et al. employed baseflow, which was obtained by using the partial duration series approach, to calibrate and validate the SWAT model [19]. Ferket et al. also used baseflow obtained by a physically based digital baseflow filter to calibrate and validate two hydrological models: the Hydrologiska Byråns Vattenavdelning (HBV) and probability-distributed model (PDM) [20]. This shows that baseflow has been widely used to evaluate the characteristics of water resources in river basins due to being less affected by the spatial variability of rainfall, temperature, and solar radiation. In recent decades, hydrologists have carried out research on baseflow, and new baseflow segmentation methods have emerged, but thus far, there is still no universally accepted method [21–23]. Li et al. found that the Lyne-Hollick method was superior to the two-parameter digital-filtering algorithm by coupling the surface water and groundwater model and recursive digital-filtering technique [24]. Chapman developed an algorithm that can be used for baseflow division in intermittent rivers [25]. Aksoy et al. used the nonlinear baseflow segmentation model to segment the base flow and then revealed evident seasonal behavior of base flow [26].

In 2019, ecological protection and high-quality development in the Yellow River Basin were promoted as a national strategy, marking a new historical period for its economic and social development [27]. Thus, in this context, it is of great significance to study the law of time scale evolution for the smooth development of ecological protection. To date, there is still no accepted method to measure baseflow processes, which can only separate baseflow from runoff using baseflow segmentation methods. In addition to the uncertainty from the watershed partitioning scheme, baseflow segmentation method uncertainty also affects hydrological processes in the calibration and validation of hydrological model parameters using baseflow components. Previous research studies have shown that the influence of watershed partitioning schemes and baseflow segmentation methods has a significant impact on the performance of hydrological models in different periods but they have not considered the impact of their simultaneous existence [28–31]. Consequently, there is a lack of understanding of the combined and interactive contributions of different uncertainty sources to the performance of hydrological models in different periods. In addition, this problem is compounded by the fact that the base flow segmentation method operates solely on the total streamflow hydrograph without considering the potential impacts of physical catchment characteristics. However, by considering the hydrological processes driving baseflow, one might expect that physical catchment characteristics have a significant impact on baseflow. For example, if the rainfall rate over a dry catchment with sandy soils is smaller than the rate of infiltration, direct runoff from the surface will be very small, and the baseflow contribution to streamflow will be significant. However, at present, it is difficult to assess this.

For the above reasons, the objective of this paper was to develop a global sensitivity analytical framework to quantitatively explore the influence of uncertainty in watershed partitioning schemes and baseflow segmentation methods on hydrological simulation. The main steps of this study included (i) based on the topographic model (TOPMODEL), PSO was used to calibrate the hydrological model parameters of the watershed partitioning scheme, and the objective function values were evaluated; (ii) three baseflow segmentation methods (HYSEP, IUKIH and Lyne-Hollick) were used to separate the baseflow from hydrological simulation processes under different watershed partitioning schemes in the source area of the Yellow River; and (iii) finally, using the variance decomposition method based on the subsampling method, we quantitatively evaluated the individual and interactive uncertainty impacts of the watershed partitioning scheme and the baseflow segmentation methods on base processes in different periods. The results of this study are helpful for deeply understanding the impact of uncertainty on water resources.

## 2 Study area and data

The Yellow River is the "Mother River" of Chinese people and is the water supply for approximately 140 million people (in more than 50 major cities). Agricultural irrigation is extensive and the basin area is 16 × 10^5^ km^2^. The river spans the Qinghai-Tibetan Plateau, Loess Plateau and North China Plain and finally flows into the Pacific Ocean, with a total length of 5464 km [32, 33]. The basin is dominated by an alpine semihumid climate. The temperature and precipitation decrease from the southeast to the northwest. The annual average precipitation is 450 mm, mainly from June to October. Base flow is an important part of river runoff. Meanwhile, as the hinterland of the Qinghai Tibet Plateau, the source area of the Yellow River, known as the "Chinese water tower", plays an important role in the development and utilization of natural ecology and water resources in the lower reaches of the Yellow River. In addition, the base discharge of the Yellow River accounts for about 44% of the river runoff, and the ratio of the multi-year average base discharge to the river runoff in the source area of the Yellow River above Tangnaihai is as high as 65.2%.

**Fig 1:**
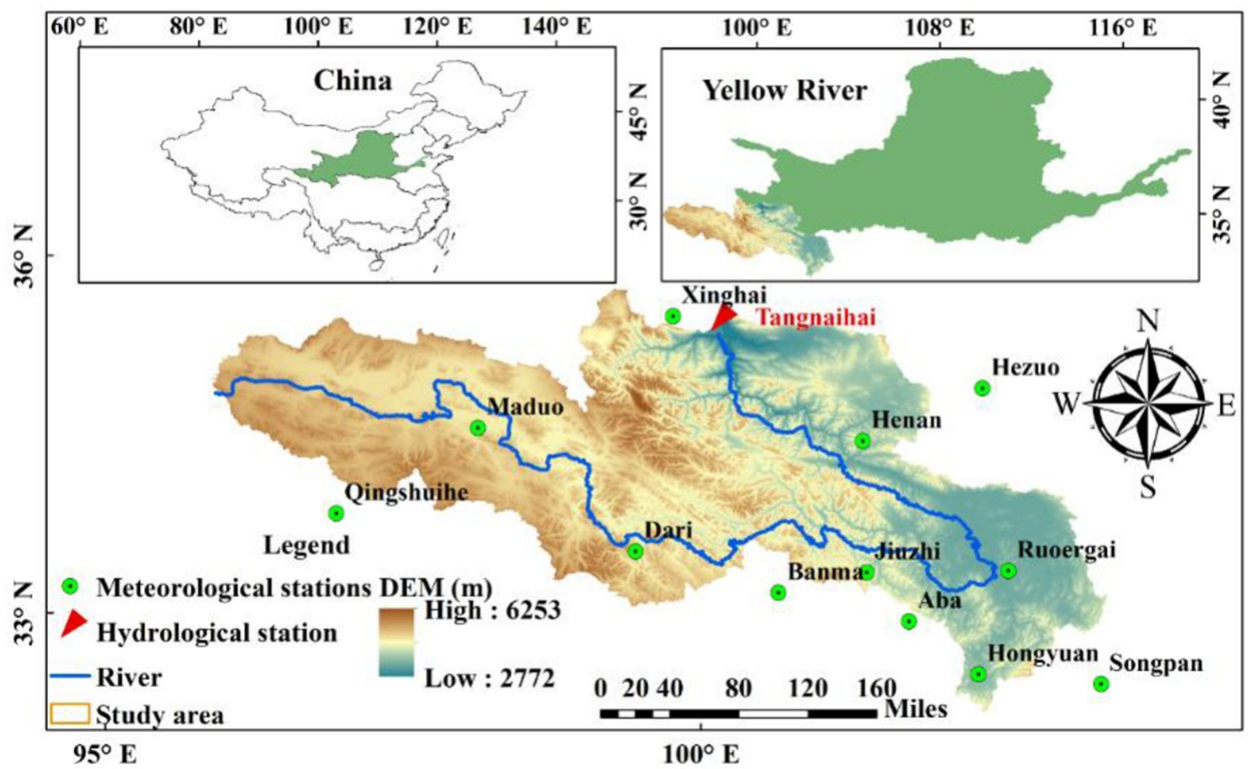
Geographic location of the basin and the spatial distribution of hydrometeorological stations.

**Fig 2:**
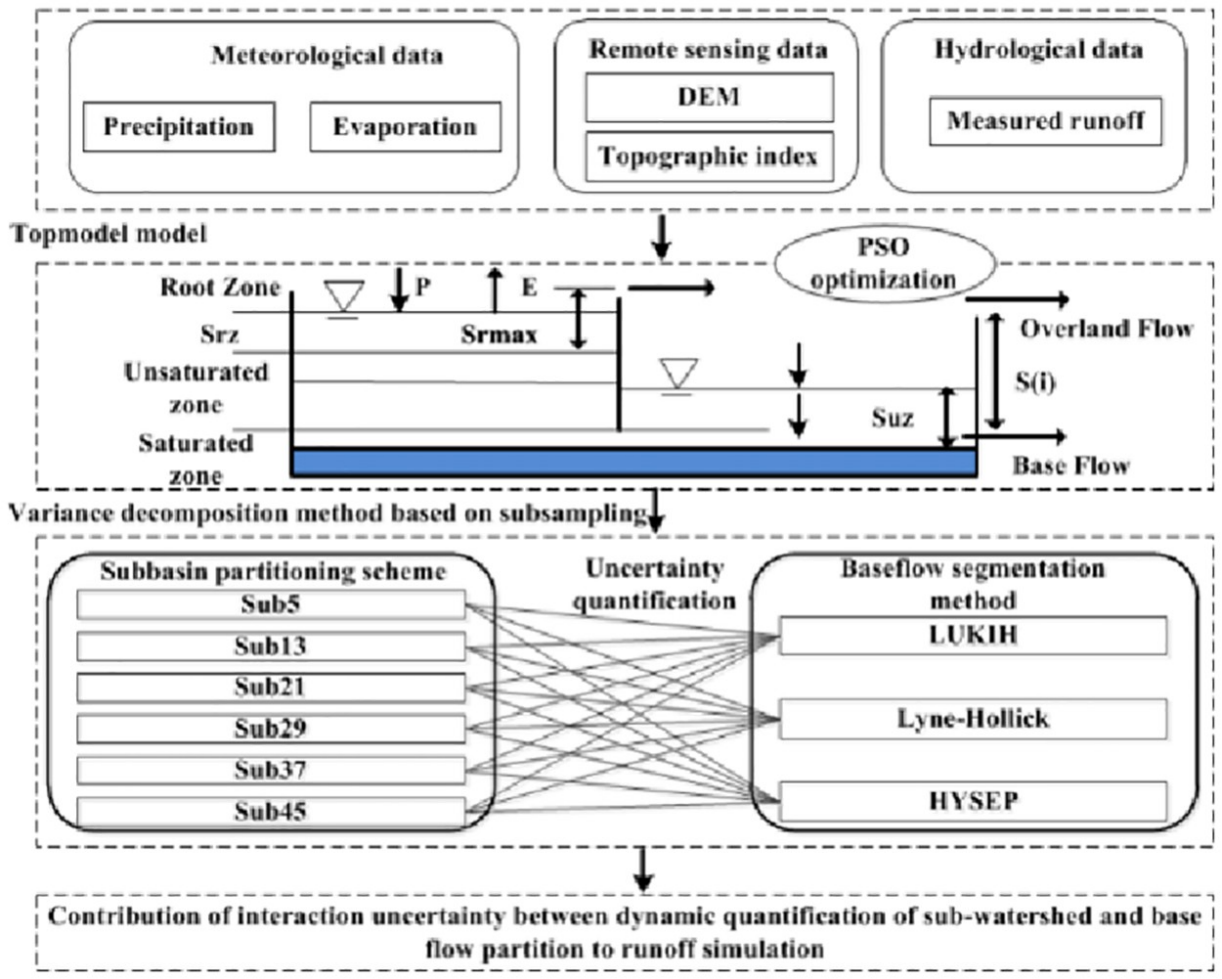
Flowchart of the methods used in this study.

The input data of the TOPMODEL hydrological model include digital elevation model, digital, terrain index and hydrometeorological data. The digital elevation model (DEM) used is the 90 m space shuttle radar terrain mission, which came from a geospatial data cloud (http://www.gscloud.cn/sources/accessdata/305?pid=302). Except for hydrometeorological data, other data were obtained by ArcGIS software. For meteorological data, the average daily precipitation, temperature, relative humidity, solar radiation and wind speed of 12 meteorological stations in the basin from 2006 to 2012 were selected, and the potential evaporation of each subbasin was calculated by using the Tyson polygon. Hydrological data and meteorological data were consistent over time. Fig 1 shows the geographic location of the basin and the spatial distribution of meteorological stations. Fig 2 shows a flowchart of the methods used in this study.

## 3 Hydrological model and methods

### 3.1 TOPMODEL model

Topmodel is a semidistributed hydrological model proposed by Beven and Kirkby in 1979 [34]. It is based on the variable source principle of the topographic index and uses soil moisture content to estimate the size and location of the source area. The model is simplified based on the following three important assumptions: (1) there is a stable saturated layer of water supply in the basin; (2) soil water conductivity and water deficit decrease exponentially; and (3) the hydraulic gradient is approximately the same as the topographic slope on the saturated area of the basin, according to Darcy’s law.

### 3.2 Model calibration method

Particle swarm optimization (PSO) is a stochastic algorithm for solving optimization problems. It has the advantages of less parameter setting, simple and easy operation, and room for improvement, and it is widely used in scientific research and engineering applications [35–37].

Each solution to the optimization problem represents a particle, which flies at a certain speed in the *n*-dimensional search space, and the fitness function is used to identify good or bad particles [38]. PSO, with its advantages of easy implementation, high accuracy and fast convergence speed, has been favored by academic circles and has been applied to many practical problems, including multiobjective optimization, signal processing, neural network training, and other fields [39, 40]. Thus, we used PSO to optimize the TOPMODEL model parameters in this paper. The particles dynamically adjust the flight speed according to their flight experience and the flight experience of other particles to obtain the best solution. The standard PSO algorithm can be described as follows: Assuming that the search space is d-dimensional, there are *Np* particles in the population, then the position of particle *i* in the group is represented as a *d*-dimensional vector *X*_*i*_ = (*X*_*i1*_,*X*_*i2*_,…,*X*_*id*_)^T^, the particle velocity can be expressed as another *d*-dimensional vector V_i_ = (*V*_*i1*_,*V*_*i2*_,…,*V*_*id*_)^T^, and then the velocity and position update of particle *i* can be obtained by the following formula.

$$v_{ij}(t+1)=\text{w}\times v_{ij}(t)+c_{1}rand1_{ij}\times (pbest_{ij}(t)-x_{ij}(t))+c_{2}rand2_{ij}\times (pbest_{ij}(t)-x_{ij}(t))$$ (1)

$$x_{ij}(t+1)=x_{ij}(t)+v_{ij}(t+1)$$ (2)

where t represents the number of particle update iterations, w represents the inertia coefficient, *c*_*1*_ and *c*_*2*_ are acceleration constants, *rand1* and *rand2* represent two independent random numbers uniformly distributed in the interval [0,1], and pbest represents the "best" position that particle i has experienced in the tth generation.

### 3.3 Baseflow segmentation method

The baseflow segmentation method is an important tool for separating baseflow and surface flow from runoff, which has received considerable attention from domestic and foreign scholars in recent years [41–44]. To simplify the baseflow segmentation process, baseflow segmentation methods are a powerful tool for segmenting the baseflow and surface flow of runoff. The widely used baseflow segmentation methods mainly include smoothing minimum (UKIH) [44], digital filtering [45] and the HYSEP method [46]. Taking into account the advantages and disadvantages of the baseflow segmentation method, three methods, the filtering smoothing minimum method (IUKIH), single parameter digital filtering (Lyne-Hollick) and fixed interval method (HYSEP), are selected to segment the runoff simulation process under different subbasin partitioning schemes in this paper. The performance of recursive digital filters is also affected by one or more user-defined parameters, which are used to change the amount of attenuation in the low/high-frequency domain of the flow spectrum and therefore have an impact on the obtained baseflow hydrograph. Thus, the HYSEP, IUKHH and Lyne-Hollick methods are data-filtering algorithms. To reduce the uncertainty of the algorithm results, we only utilized these three algorithms.

### 3.4 Variance decomposition method based on subsampling

The multivariate variance decomposition method was proposed by Bosshard in 2013 [47]. It has been successfully applied to examine the effects of individual variables and their interactions on dependent variables. This method has been widely used to explore the degree of contribution of uncertainty between different sources. Therefore, this method explores the uncertainty impact of subbasin segmentation schemes and base-flow segmentation methods on runoff simulation. The main steps are as follows.

Suppose *Y* is a variable whose two influencing factors are *A* and *B*, where *A* and *B* contain *M* and *K* samples, respectively; then, the combination of *A* and *B* produces the sum of *M* × *K* samples.


$$Y^{j,k}=A^{j}+B^{k}+AB^{j,k}$$ (3)

**Fig 3:**
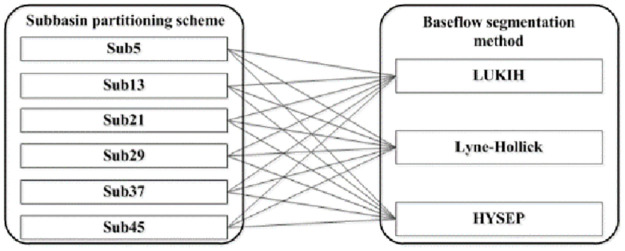
Combination process of the subbasin partitioning scheme and baseflow segmentation method.

In this paper, *A* represents the *j*th subbasin partitioning scheme, *B* represents the *k*th baseflow segmentation method, and *AB* represents the interaction influence between the two. The process of combining the subbasin partitioning scheme and baseflow segmentation method is shown in Fig 3.

The influence level of each uncertainty source depends on the number of samples. To solve this problem, Bosshard proposed a subsampling method to eliminate the influence of the number of samples. The main process of this method is that for iteration *i*, two samples are randomly selected from all samples of a factor, such as the samples in watershed partitioning schemes A and *g(h*, *i*), which are used instead of *j* in *Y*^*j*,*k*^. The total number of subsamplings is 15, i.e., *I* = 15, in this paper, and its combination matrix is as follows.


$$\text{g}=\left(\begin{array}{llllllllll} 1 & 1 & \cdots & 1 & 2 & 2 & \cdots & 4 & 4 & 5\\ 2 & 3 & \cdots & 6 & 3 & 4 & \cdots & 5 & 6 & 6 \end{array}\right)$$ (4)

The total variance contribution (*SST*) can be decomposed into the contribution of *A* and *B* and the contribution of the combination of Factors *A* and *B*, and its contributions are expressed as *SSA*, *SSB*, and *SSI*, respectively.

SST=SSA+SSB+SSI (5)

$$SST=\sum_{h=1}^{H}\sum_{k=1}^{K}{\left(M^{g(h,i),k}-M^{g(o,i),o}\right)}^{2}$$ (6)

$$SSA_{i}=K\times \sum_{h=1}^{H}{\left(M^{g(h,i),o}-M^{g(o,i),o}\right)}^{2}$$ (7)

$$SSB_{i}=H\times \sum_{k=1}^{K}{\left(M^{g(o,i),k}-M^{g(o,i),o}\right)}^{2}$$ (8)

$$SSI_{i}=\sum_{h=1}^{H}\sum_{k=1}^{K}{\left(M^{g(h,i),k}-M^{g(h,i),o}-M^{g(o,i),k}-2\cdot M^{g(o,i),o}\right)}^{2}$$ (9)

where *SST* represents the contribution of total variance, *SSA* represents the variance contribution of the subbasin partitioning scheme, *SSB* is the variance contribution for the baseflow segmentation method, *SSI* represents the contribution of the partitioning scheme and baseflow segmentation method interaction between the two, and the symbol o represents the mean flow in *i* subsamples. *H* and *K* represent the number of subbasin partitioning schemes and the baseflow segmentation method, respectively. In this paper, *H* = 6 and *K* = 3. The variance contribution rate of different factors is calculated by the following formula.

$$\eta_{subbasin}=\frac{1}{I}\sum_{i=1}^{I}\frac{SSA_{i}}{SST_{i}}$$ (10)

$$\eta_{baseflow}=\frac{1}{I}\sum_{i=1}^{I}\frac{SSB_{i}}{SST_{i}}$$ (11)

$$\eta_{interaction}=\frac{1}{I}\sum_{i=1}^{I}\frac{SSI_{i}}{SST_{i}}$$ (12)

where *η* is a number between 0 and 1, and they represent the contribution degrees of 0% and 100%, respectively.

### 3.5 Evaluation index

Four statistical metrics, including the Kling-Gupta coefficient (*KGE*), Nash efficiency coefficient (*NSE*), correlation coefficient (*R*^2^) and relative error (*RE*), were used to evaluate the model performance [48, 49]. Taking into account the advantages of the *KGE* indicator in model evaluation performance [50], this paper selected this as the objective function and uses other indicators to evaluate model simulation accuracy.

$$KGE=1-\sqrt{{\left(r-1\right)}^{2}+{\left(\alpha -1\right)}^{2}+{\left(\beta -1\right)}^{2}}$$ (13)

$$NSE=1-\frac{\sum_{i=1}^{n}{\left(Q_{obs,i}-Q_{\text{sim},i}\right)}^{2}}{\sum_{i=1}^{n}{\left(Q_{obs,i}-{\bar{Q}}_{obs}\right)}^{2}}$$ (14)

$$R^{2}=\frac{\sum_{i=1}^{n}{\left[(Q_{sim,i}-{\bar{Q}}_{sim})(Q_{obs,i}-{\bar{Q}}_{obs})\right]}^{2}}{\sum_{i=1}^{n}{\left(Q_{obs,i}-{\bar{Q}}_{obs}\right)}^{2}\sum_{i=1}^{n}{\left(Q_{sim,i}-{\bar{Q}}_{sim}\right)}^{2}}$$ (15)

$$|Re|=\frac{{\bar{Q}}_{sim}-{\bar{Q}}_{obs}}{{\bar{Q}}_{obs}}\times 100\%$$ (16)

where *r*, *α* and *β* represent the correlation coefficient, relative dispersion degree and mean deviation of the simulated and measured flows, respectively, and *Q*_*sim*,*i*_ and *Q*_*obs*,*i*_ represent the *i*th simulated and observed flow, respectively. ${\bar{Q}}_{sim}$ and ${\bar{Q}}_{obs}$ represent the average values of the measured flow and simulated flow, respectively. *n* represents the length of the runoff series.

## 4 Results and analysis

Hydrological models play an important role in simulating hydrological processes of a watershed, and the basic steps for successfully constructing a hydrological model are dividing a catchment basin into different subbasin response units and then calibrating and validating the hydrological model parameters. Beven and Binley showed that identical model results can be obtained using different parameter combinations, which indicates that similar streamflows can be modeled with different combinations of surface runoff and baseflow [14]. For example, Rouhani et al. employed baseflow, which was obtained by using the partial duration series approach, to calibrate and validate the SWAT model [19]. Ferket et al. also used baseflow obtained by a physically based digital baseflow filter to calibrate and validate HBV and PDM hydrological models. However, the randomness of subbasin division and the selection of baseflow segmentation methods may lead to significant differences in runoff simulation accuracy [20]. Thus, quantifying the effects of subbasin partitioning schemes and baseflow segmentation methods on runoff simulation has important practical significance.

### 4.1 Spatial distribution characteristics of subwatershed division

**Fig 4:**
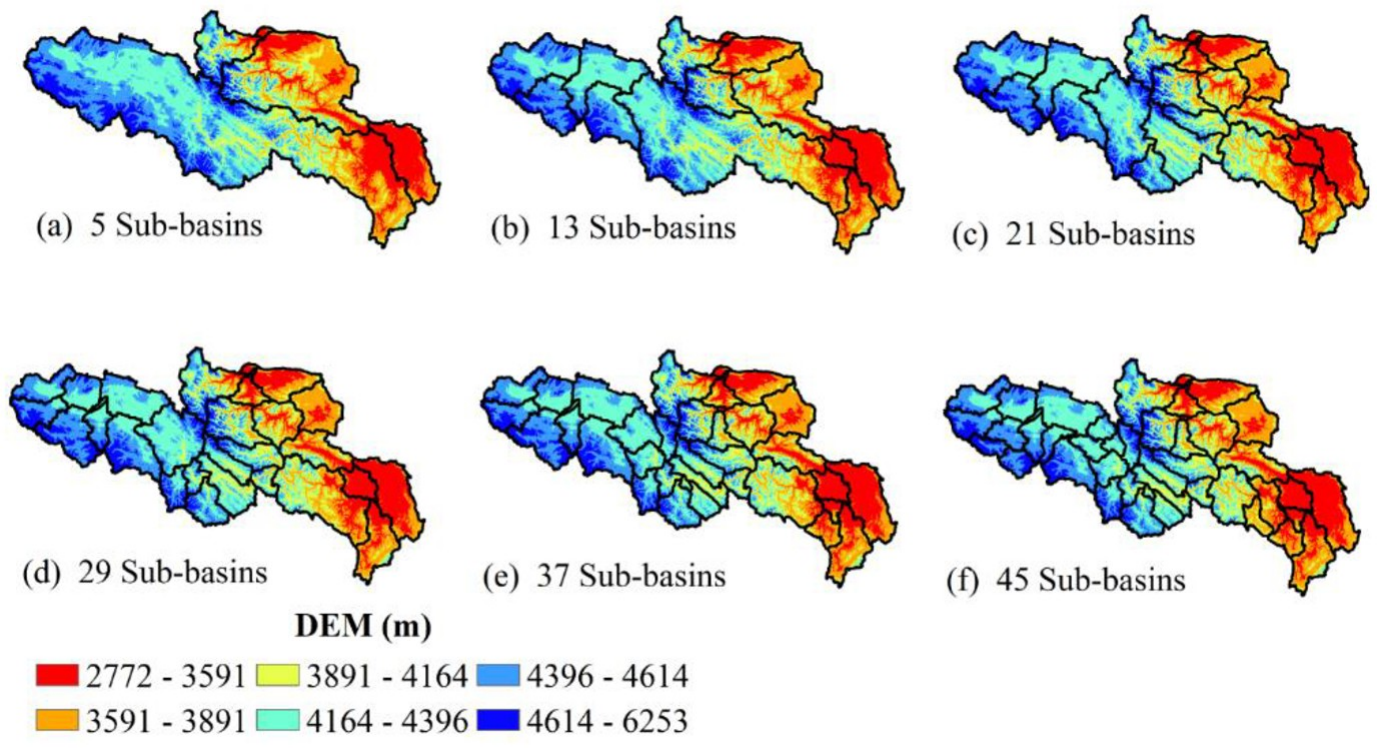
Spatial distribution information of the subbasin partitioning scheme under different catchment area thresholds (the base map for this figure originated from NASA earth observatory (public domain)).

Fig 4 represents the spatial distribution of the subbasin partitioning scheme under different catchment area thresholds in the source region of the Yellow River. The elevation of the basin gradually decreases from the west to east, and the elevation range of the basin is between 2772 and 6553 m.

Table 1 shows the parameter values of the TOPMODEL model under different subbasin division schemes. As seen from Table 1, under different subbasin division schemes, the differences in the parameter values of *T*_*0*_, *s*_*zm*_, *T*_*d*_, and *SR*_*max*_ were small, but the differences in the parameters *R*_*v*_, *CHV* and *SR*_*0*_ were large. This indicates that the subbasin division has little impact on the time lag coefficient of gravity drainage, the maximum water storage of the root zone and the initial water content of the root zone but has a significant impact on the effective speed of river confluence and river width.

**Table 1:** Parameter values of the TOPMODEL model under different subbasin division schemes.

Subbasins	*T*_*0*_	*s*_*zm*_	*T*_*d*_	*SR*_*max*_	*R*_*v*_	*CHV*	*SR*_*0*_
5	4.05	0.06	15.61	0.05	4165.60	5479.64	376.28
13	5.56	0.05	15.09	0.06	4358.30	6899.54	242.81
21	5.25	0.05	15.70	0.05	4352.04	5933.44	262.83
29	4.55	0.05	15.60	0.06	4441.20	5770.10	239.25
37	4.65	0.05	17.32	0.06	5663.59	5689.59	213.17
45	5.17	0.05	14.56	0.05	5309.38	5824.09	275.06

### 4.2 Model performance evaluation

For the six subbasin partitioning schemes, the data from January 1, 2005, through December 31, 2005 were used for model warm-up, the data from January 1, 2006, through December 31, 2009 were used for model calibration, and the data from January 1, 2010, through December 31, 2012 were used later for model validation.

**Fig 5:**
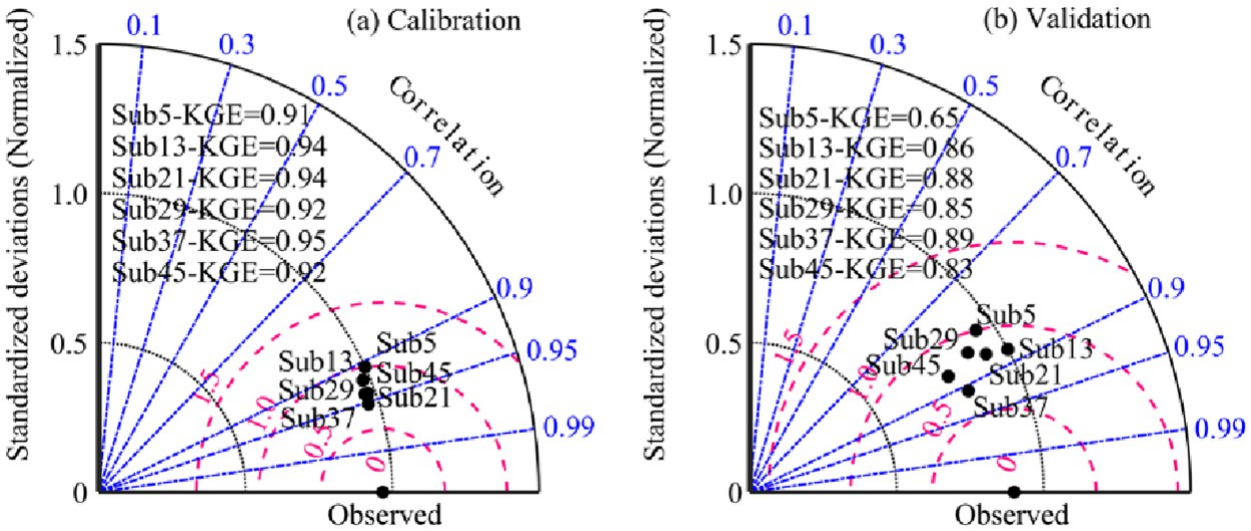
Taylor plot results for calibration and validation periods under different subbasin partitioning schemes.

This paper selected the *KGE* index as the objective function of the TOPMODEL model calibration and validation periods. Fig 5 represents the Taylor plot results for calibration and validation periods under different subbasin partitioning schemes. A number of conclusions can be obtained from Fig 5. In addition to Sub 29, the accuracy of the TOPMODEL hydrological model performance increased gradually with the increase in the number of subbasins partitioned up to a certain threshold and then decreased. The number of subbasins partitioned had a small influence on the standard deviation and root mean square error in the calibration period but had a greater impact during the validation period. In the calibration and validation periods, the objective function values (*KGE*) in the Sub5, Sub13, Sub21, Sub29, Sub37 and Sub13 scenarios were 0.91 and 0.65, 0.94 and 0.86, 0.94 and 0.88, 0.92 and 0.82, 0.95 and 0.89, and 0.92 and 0.83, respectively. In addition, the *KGE* in model validation was lower than that in validation but was always more than 0.65 for all subbasin partitioning schemes.

**Fig 6:**
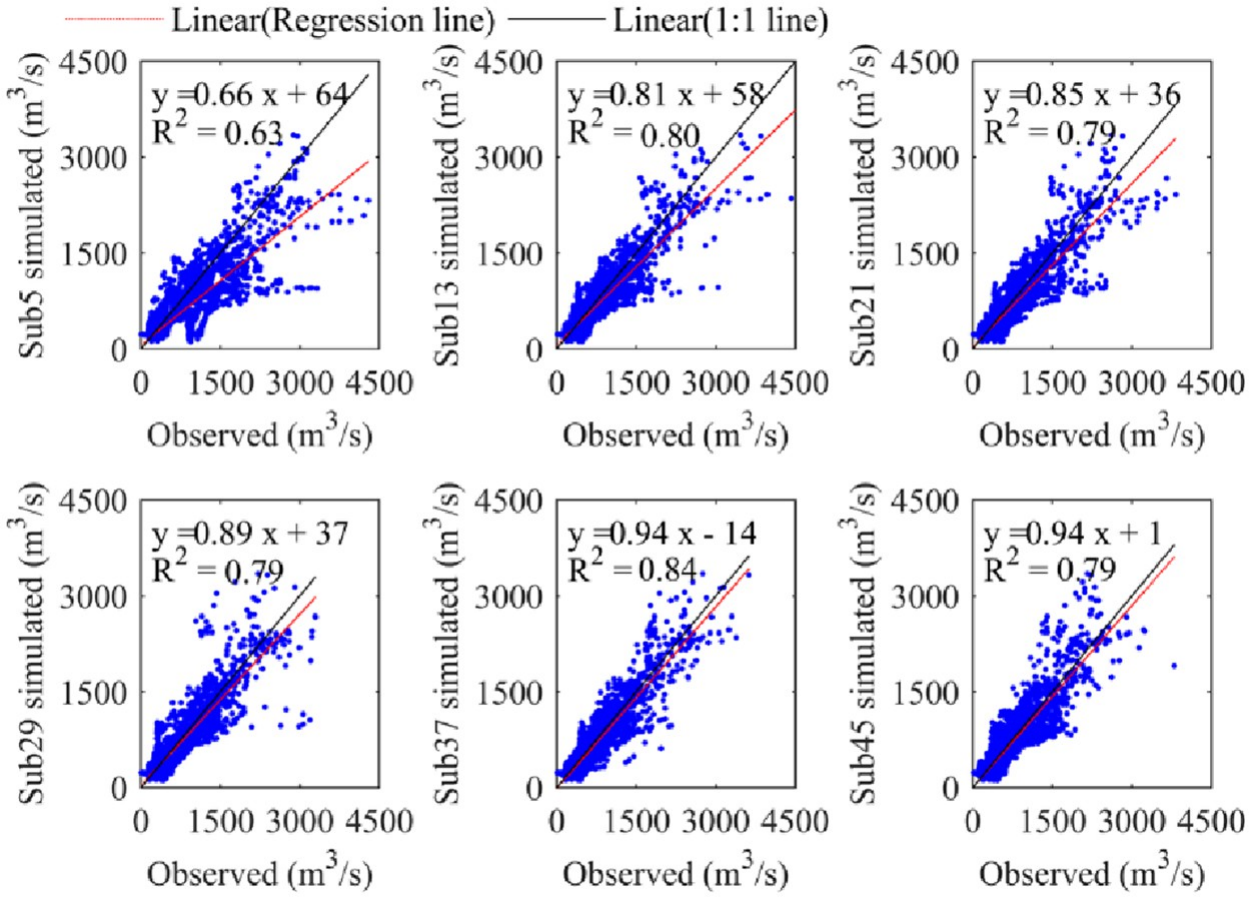
Scatter plot results of the observed and model-simulated streamflow under different subbasin partitioning schemes.

Fig 6 represents the observed and model-simulated daily streamflow scatter plot results, in which the observed streamflow is plotted along the *x*-axis and the simulated model is plotted along the *y*-axis. It is worth pointing out that the farther the linear regression slope line is from the 1:1 line, the worse the model simulation accuracy. In Fig 6, when only the correlation coefficient is considered, the difference in model simulation accuracy is small, but considering the correlation coefficient and the linear regression slope simultaneously, we can see that the model simulation accuracy under different subbasin partitioning schemes are significantly different, e.g., the Sub5 linear regression line is far from the 1:1 line and located below it, indicating that the model-simulated streamflow is much lower than the observed streamflow for most months, while the Sub37 linear regression line is closer to the 1:1 line, which indicates that the TOPMODEL model has poor simulation accuracy in the Sub5 scenario and has good simulation accuracy in the Sub37 scenario. In addition, in the Sub37 scenario, the simulated discharge is obviously underestimated. In addition, as the number of subbasin partitions increases, the linear regression line gradually approaches the 1:1 line up to a certain threshold and then decreases. Overall, the number of subbasin divisions has a significant impact on the model simulation accuracy.

### 4.3 Effect of subbasin partitioning scheme uncertainty on the hydrological simulation process

**Fig 7:**
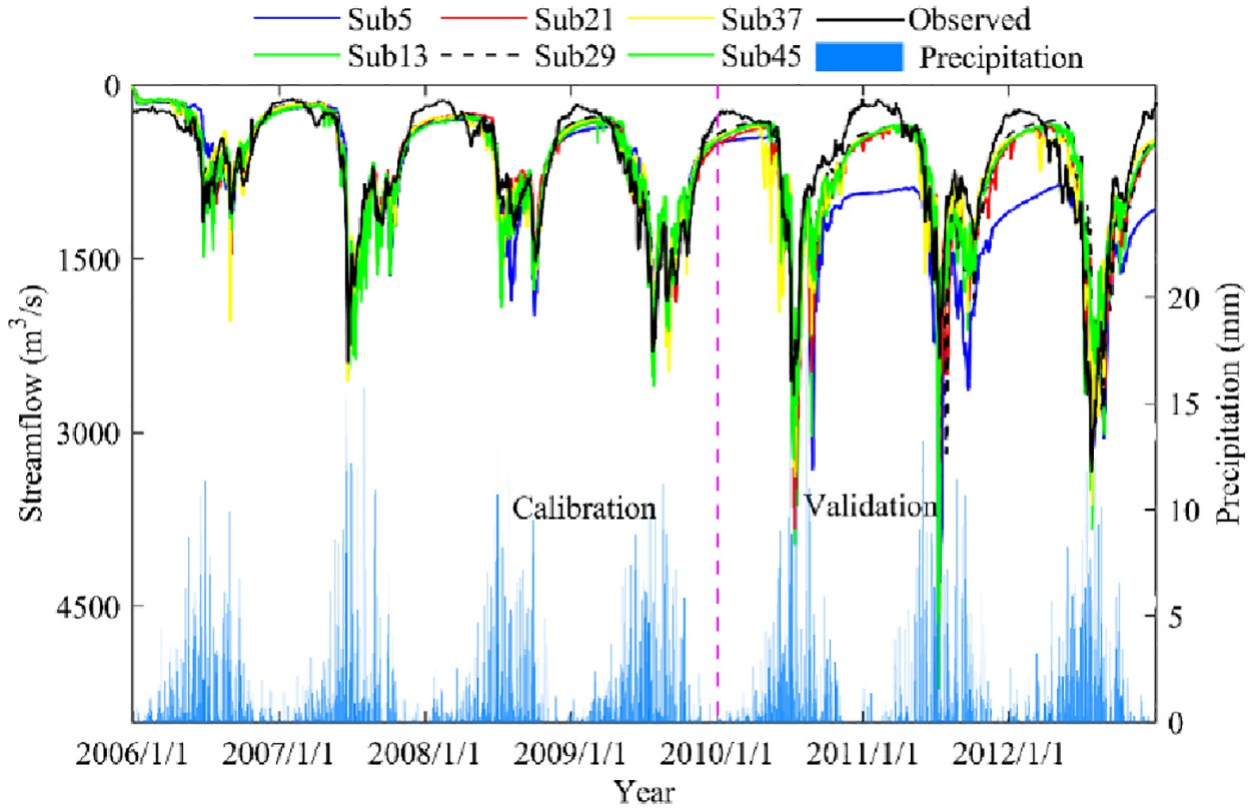
Results of the model performance evaluation in calibration and validation periods.

From the previous section, we can see that the performance evaluation results of different subbasin partitioning scheme models are obviously different. To further explore the influence of subbasin partitioning scheme uncertainty on hydrological processes in different characteristic periods, we conducted statistical analysis from the perspective of hydrological processes and monthly streamflow values (calibration and validation). Fig 7 shows the results of runoff simulation in calibration and validation periods under different subbasin partitioning schemes. A number of important conclusions can be obtained from this plot. First, the results of the modeled discharge are similar to the observed discharge, which indicates the better accuracy of runoff simulation under different subbasin partitioning schemes. However, the process of runoff simulation showed significant differences in different characteristic periods, e.g., the data from October 1, 2010, through April 31, 2011 and from June 1, 2011, through October 31, 2011. Again, the runoff simulation accuracy in the calibration period was better than that in the validation period. In addition, the precipitation in the basin was mostly concentrated in the flood period (June to September), the maximum daily precipitation was as high as 25 mm/day, the precipitation in the nonflood period was less, and the daily precipitation was less than 0.5 mm/day. Overall, all subbasin partitioning scheme simulations yielded very good results.

**Fig 8:**
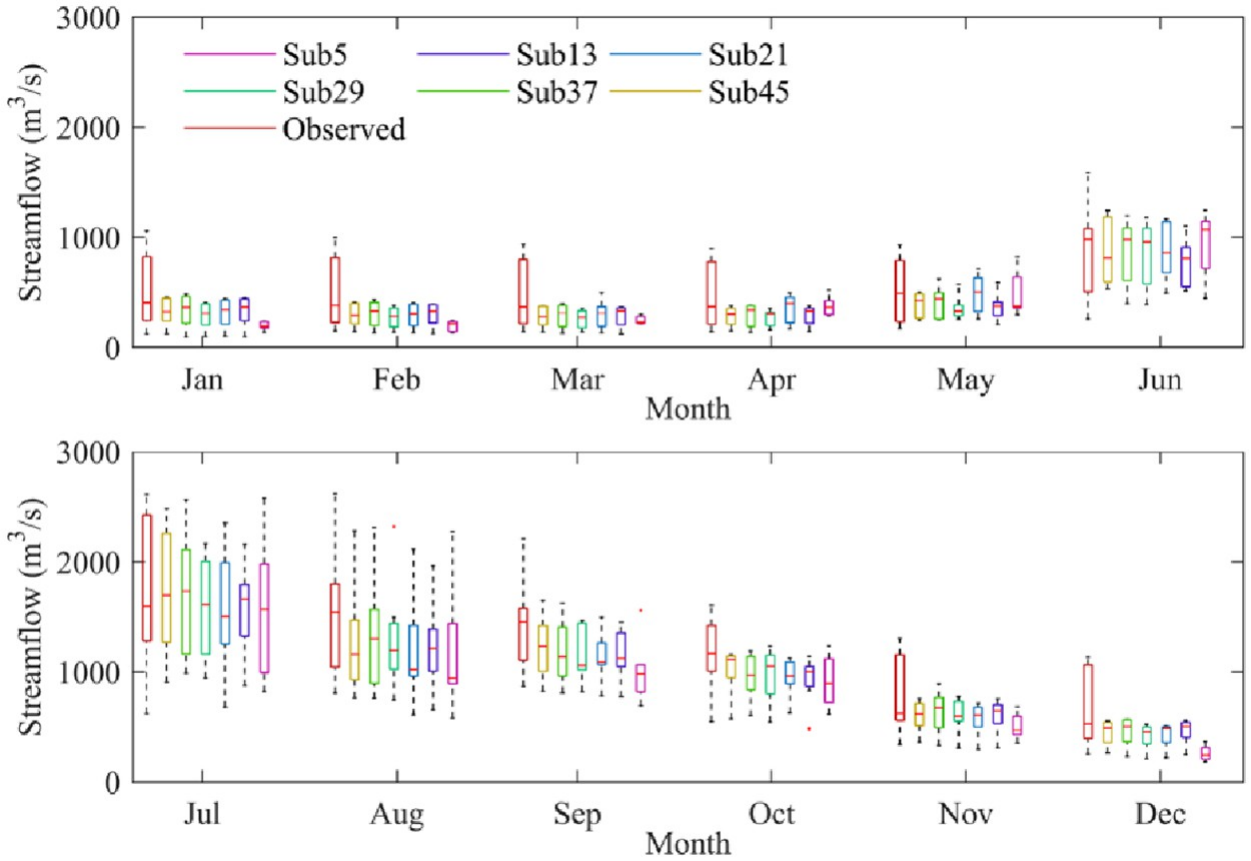
Difference between the monthly modeled discharge and the measured discharge under subbasin partitioning schemes.

For the different subbasin partitioning schemes, the TOPMODEL model performance gradually improved with the increase in the subbasin division number, while the improvement began to decline when the subbasin division number exceeded a threshold, i.e., Sub37 in this paper. The boxplots of the streamflow between the observed and model-simulated streamflow under different subbasin partitioning schemes for different months are shown in Fig 8. In Fig 8, the subbasin division uncertainty led to a significant difference in simulated streamflow for different months. In the dry season, the simulated streamflows obtained by different subbasin partitioning schemes were all smaller than the measured streamflows, indicating that the simulated streamflow was obviously underestimated for most months; however, the simulated streamflow was close to the measured streamflow during the wet season, especially in June and July. Moreover, the subbasin division uncertainty had less impact on simulated streamflow during the dry season and had a significant impact in the wet season; for example, the subbasin division uncertainty caused the difference between the median of the simulated streamflow to be as high as 213.09 m^3^/s in August but only 107.19 m^3^/s in January. In addition, this also shows that the model performance in the Sub5 scenario had the worst simulation accuracy, which can sometimes result in overestimations and sometimes underestimations of the simulated discharge, such as in January to February and September to December. Moreover, the error range and trend of the simulated streamflow of subbasin division schemes were basically consistent with the measured streamflow during the wet season, but the dry season was the opposite.

### 4.4 Effect of baseflow segmentation method uncertainty on hydrological simulation

In recent decades, a large number of baseflow segmentation methods have been developed [16–18], and it is possible to use continuous assessments of baseflow for the calibration and validation of hydrological models. However, there is a large amount of uncertainty in choosing baseflow segmentation methods. Thus, it is important to explore the effect of baseflow segmentation method uncertainty on the hydrological simulation process.

**Fig 9:**
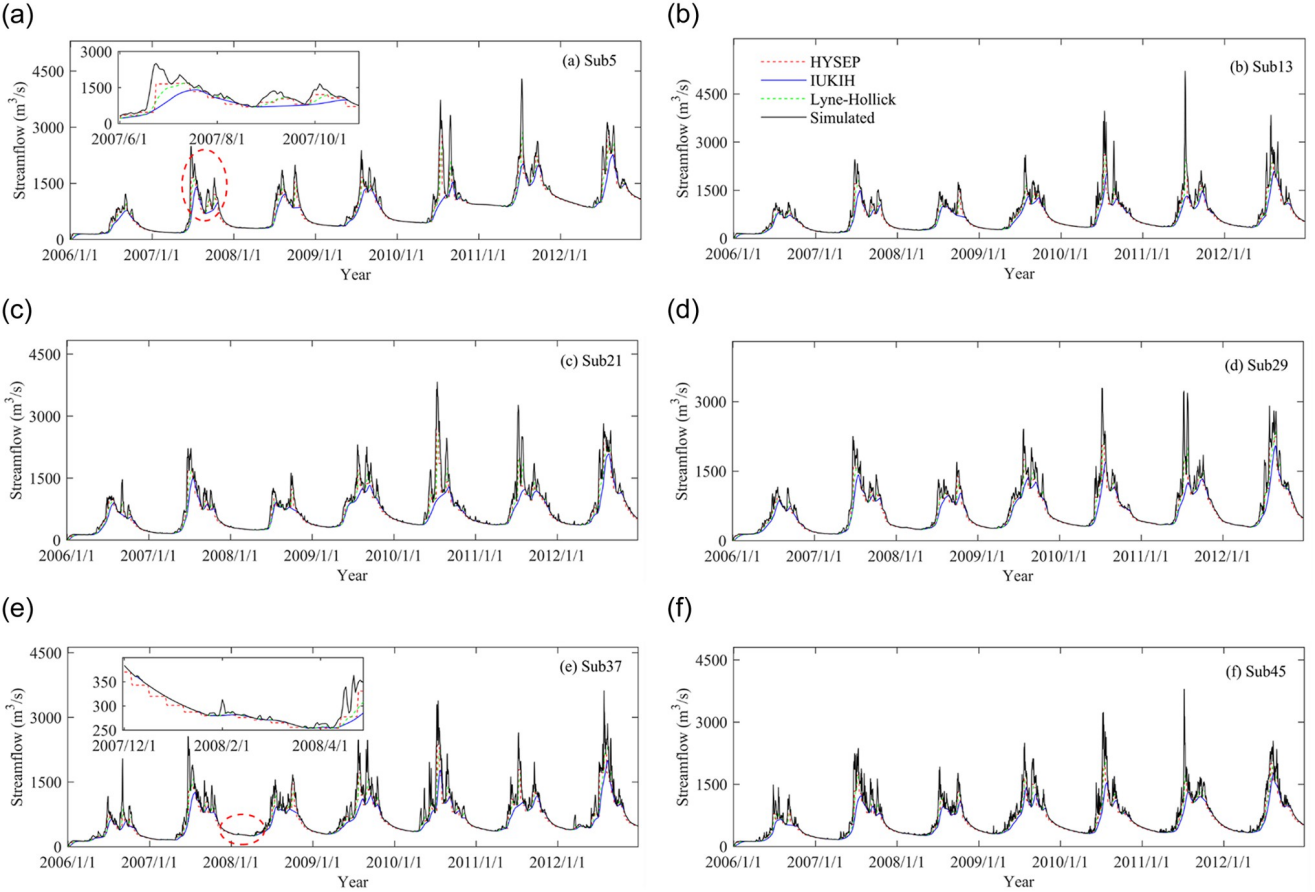
Effect of baseflow segmentation method uncertainty on hydrological processes under subbasin partitioning schemes.

The plots of the effect of baseflow segmentation method uncertainty on hydrological processes under subbasin partitioning schemes are shown in Fig 9. A number of conclusions can be obtained from these plots. First, the baseflow varied with the change in simulated runoff, which is more obvious for the Lyne-Hollick and IUKIH methods. Second, the baseflow values estimated by the Lyne-Hollick method and HYSEP method were obviously higher than those estimated by the IUKIH method during the wet season (see Fig 9(a) submap). Finally, the influence of the baseflow segmentation methods on the simulated streamflow is obvious during the different characteristic periods. For example, in the Sub5 scenario, the daily mean baseflows in 2007 were estimated to be 273.04 m^3^/s, 283.84 m^3^/s, and 283.92 m^3^/s using the HYSEP, IUKIH, and Lyne-Hollick methods during the dry season, respectively, while the daily mean baseflows were estimated to be 847.14 m^3^/s, 721.06 m^3^/s, and 848.92 m^3^/s, respectively, during the wet season. This indicates that the baseflow values were underestimated by the IUKIH method, especially during the wet season.

In addition, the baseflow segmentation method uncertainty has a significant impact on the annual mean streamflow values under different subbasin segmentation schemes. For example, in the Sub5 scenario, the annual mean baseflows in 2007 were estimated to be 562.58 m^3^/s, 504.38 m^3^/s, and 568.88 m^3^/s using the HYSEP, IUKIH, and Lyne-Hollick methods, respectively, but the annual mean baseflows in the Sub37 scenario were estimated to be 524.94 m^3^/s, 492.91 m^3^/s, and 549.12 m^3^/s, respectively. This also indicates that the baseflow values were underestimated by the IUKIH method, and as the model simulation accuracy increased, the difference gradually decreased.

**Fig 10:**
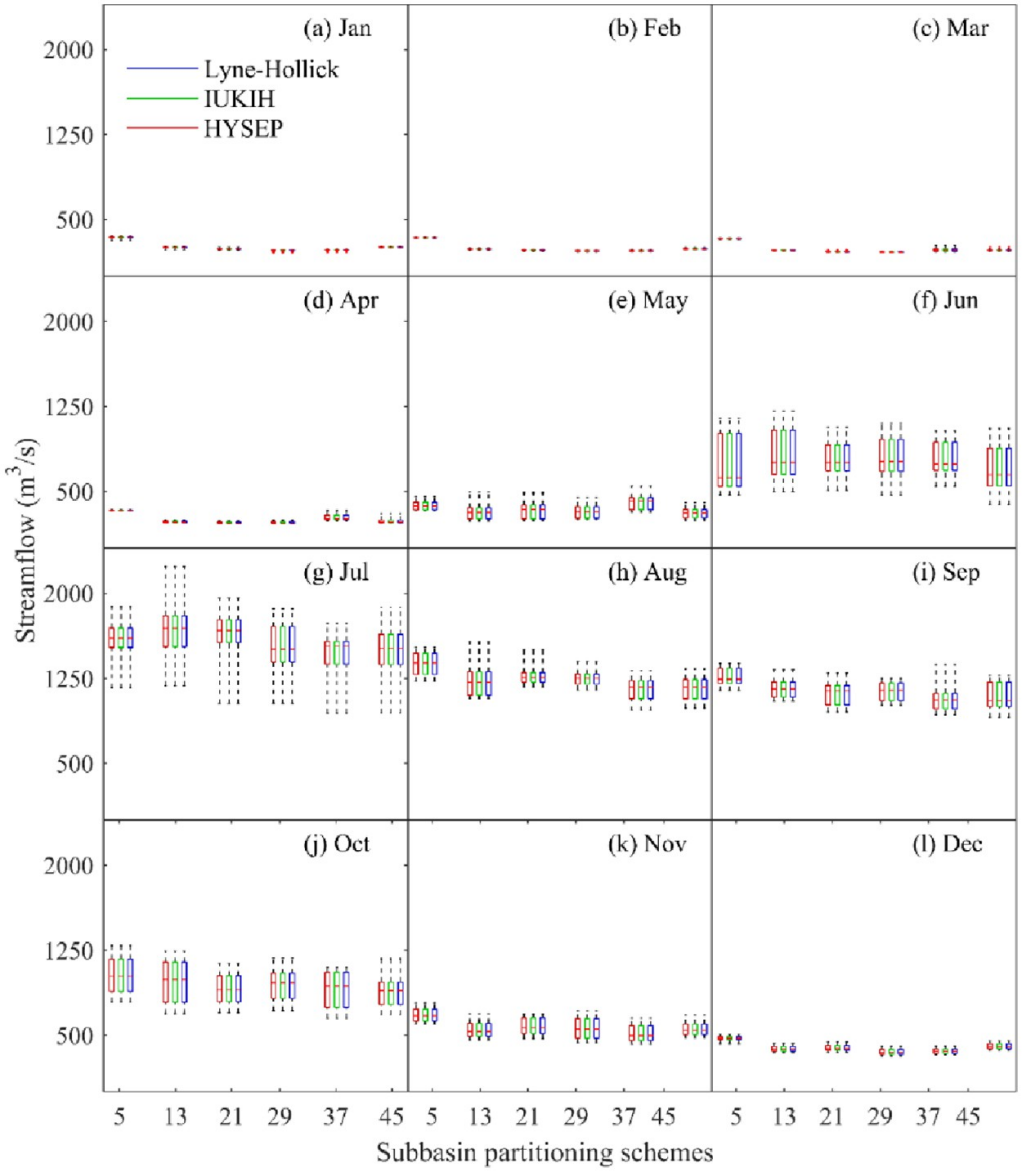
Effect of baseflow segmentation method uncertainty on streamflow in different months under subbasin partitioning schemes.

The plot of the effect of baseflow segmentation method uncertainty on runoff in different months under subbasin partitioning schemes is shown in Fig 10. The results show that the baseflow segmentation method uncertainty had less influence on runoff in different months; however, the baseflow values obtained by the HYSEP, IUKIH, and Lyne-Hollick methods under the different subbasin partitioning schemes were significantly different. The baseflow hydrographs obtained by different baseflow segmentation method change trends were basically consistent; however, as the simulated streamflow increased during the wet season, the baseflow value change error also increased, and a maximum appeared in June. In addition, as the simulated streamflow decreased during the postflood period, the baseflow value change error decreased accordingly.

Furthermore, taking May to August as an example, Fig 10 also shows that as the number of subbasin partitions increased, the median baseflow values gradually increased and then decreased, but it had a downward trend in other months. The uncertainty of subbasin partitioning schemes in August had the greatest impact on baseflow processes. The median baseflow difference was as high as 213.09 m^3^/s but was less affected in May, and the median baseflow difference was only 107.19 m^3^/s. Moreover, the distribution of baseflow values had significant differences in different months, and the baseflow values had a nonnormal distribution in June, July, and September-November but had a normal distribution in May. This finding indicates that the subbasin partitioning schemes and baseflow partitioning methods caused uneven distributions of baseflow values in the wet season.

### 4.5 Quantitative assessment of the uncertainty effects of subbasin partitioning schemes and baseflow segmentation methods on baseflow processes

Based on the above discussion, the uncertainty of subbasin partitioning and baseflow segmentation methods were significantly different on the baseflow values in different characteristic periods, and then the independence and interaction of multiple factors led to great uncertainty in the model output. How to quantify the independence and interaction of multiple source factors faces challenges. However, Bosshard proposed that a method of variance decomposition based on subsampling can successfully solve this problem, which has been widely applied [36].

**Fig 11:**
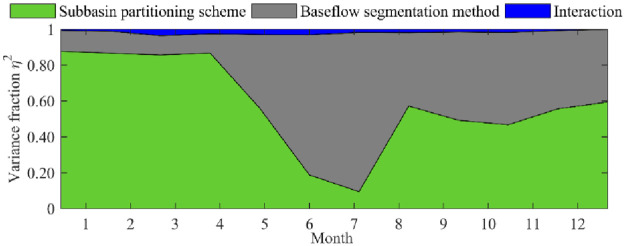
Contribution of uncertainty of the subbasin partitioning schemes, baseflow segmentation methods and their combined effects on runoff simulation.

In this paper, the variance decomposition method based on subsampling was used to quantify the influence of subbasin partitioning and baseflow segmentation methods on annual baseflow processes. It is worth noting that the error contribution in the graph is the relative value, and the interaction effect includes the contribution of the TOPMODE model. The plot of the contribution of uncertainty of the subbasin partitioning schemes, baseflow segmentation methods and their combined effects on runoff simulation is shown in Fig 11, and a number of important conclusions are as follows.

First, the contribution of uncertainty of the subbasin partitioning schemes, the baseflow segmentation methods and their interactions had significant differences in the baseflow values in different months and were especially significant during wet and dry seasons. This further verified the uncertainty influence of subbasin partitioning schemes and baseflow segmentation methods on the hydrological simulation process.

Second, in the dry season (January to March), the subbasin partitioning scheme uncertainty had the greatest impact on the simulation process, accounting for approximately 86%, the baseflow segmentation methods took second place, accounting for approximately 12%, and the combination of subbasin partitioning schemes and baseflow segmentation method uncertainty had the smallest impact, accounting for approximately 2%. In addition, in the preflood period (April to May), as the temperature rose, glaciers and snow meltwater recharged the underground baseflow values, and then the baseflow segmentation methods were further enhanced by the influence of the underground baseflow values; thus, the contribution of uncertainty of the baseflow segmentation methods gradually increased. Correspondingly, the influence of the subbasin partitioning scheme uncertainty gradually decreased.

Third, in the flood period (June to August), affected by concentrated rainfall, the uncertainty influence of the baseflow segmentation methods was dominant, with the largest impact in July, accounting for 88.24%, and the smallest in August, accounting for 46.33%. In addition, from July to August, the uncertainty influence of the baseflow segmentation methods was gradually weakened, which may have been due to the uncertainty influence of the TOMODEL hydrological model. In addition, the flood period was the flood peak period of the basin, and the contribution of subbasin division uncertainty to base flow processes increased, which is also an important finding of this paper.

Last, in the postflood period (September to December), the uncertainties in the influence of subbasin partitioning schemes, baseflow segmentation methods and their interactions were significantly different, accounting for 50.63%, 48.33% and 1.04%, respectively. The uncertain interaction influence of the subbasin partitioning schemes and the baseflow segmentation methods gradually decreased, which may be attributed to the fact that the basin is located in an alpine climate zone, and the winter temperature was relatively low, resulting in the river forming glaciers; thus, the infiltration flow decreased. Moreover, the source area of the Yellow River is located in a mountain and gully area, and the runoff in winter was very small. The subbasin division scheme and the base flow division method contributed to base flow processes. Most base flow was used to supplement river runoff; therefore, the interaction between the two was small.

## 5 Discussion

Due to the influence of different climate and underlying surface types, the base flow index of rivers in humid areas is generally low, while that in arid and semi-arid areas is high [51–53]. Subbasin partitioning schemes and baseflow segmentation methods are very important for baseflow processes. Such as, both Arabi et al. and Han et al. found that the subwatershed division scheme under hydrological model had an important impact on hydrological processes [8, 9]. Lin et al. investigated the impacts of watershed partitioning on simulation of hillslope sediment generation and its spatial variations, and found that the Hillslope sediment generation was seriously affected by watershed subdivision levels, increasing the number of sub-watersheds would decrease the modelled amount of hillslope sediment generation and increase its spatial variations [54]. Wang et al. investigated the features of PMAs (priority management areas) with differentsubdivision schemes in hydrological conditions, and found that PMAs identification can be affected by watershed subdivision with different amountsof information, and the proper increase in the number of sub-watersheds can promote the accuracy ofPMAs identification [55]. Meanwhile, the application of base flow data for hydrological model verification has achieved better results. Such as, Rouhani et al. employed baseflow, which was obtained by using the partial duration series approach, to calibrate and validate the SWAT model [19]. Ferket et al. also used baseflow obtained by a physically based digital baseflow filter to calibrate and validate two hydrological models: the Hydrologiska Byråns Vattenavdelning (HBV) and probability-distributed model (PDM) [20]. Analysis of the above studies found that the base flow process obtained by different base flow segmentation methods affects the performance of hydrological model. For example, Li et al., Chapman and Aksoy et al. found that different base flow segmentation methods obtained different evaluation results [24–26]. In general, it is very important to select appropriate and reliable base flow series as the basis for model validation. Meanwhile, the randomness of subbasin division and the subjectivity of the selection of the baseflow segmentation methods result in inconsistent runoff simulation values in different months. The combination of base flow segmentation method and TOPMODEL model provides a new opportunity to study the spatial distribution characteristics of base flow and surface runoff, and also provides a new idea for the spatialization of surface process elements. To fill this gap, we explored the influence of their interaction uncertainty on base flow processes. We found that the subbasin division scheme and base flow division method had different effects on base flow simulation in different periods. The research results have important practical significance for an in-depth understanding of water resource assessments with base flow as the recharge source.

**Fig 12:**
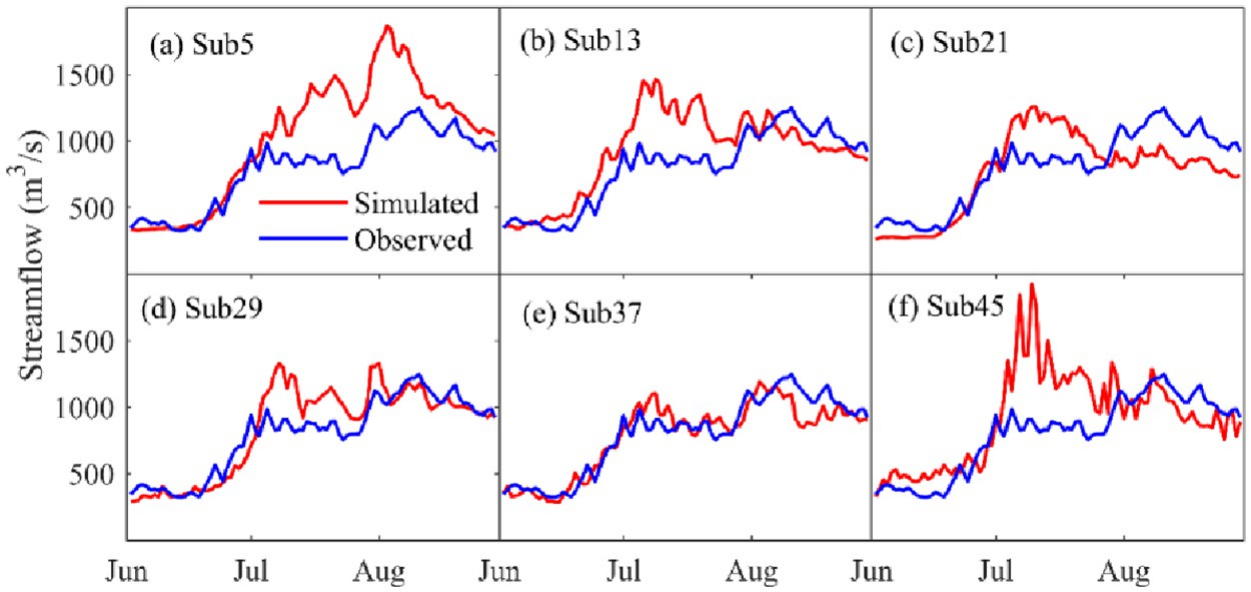
Influences of subbasin division on water resources.

**Fig 13:**
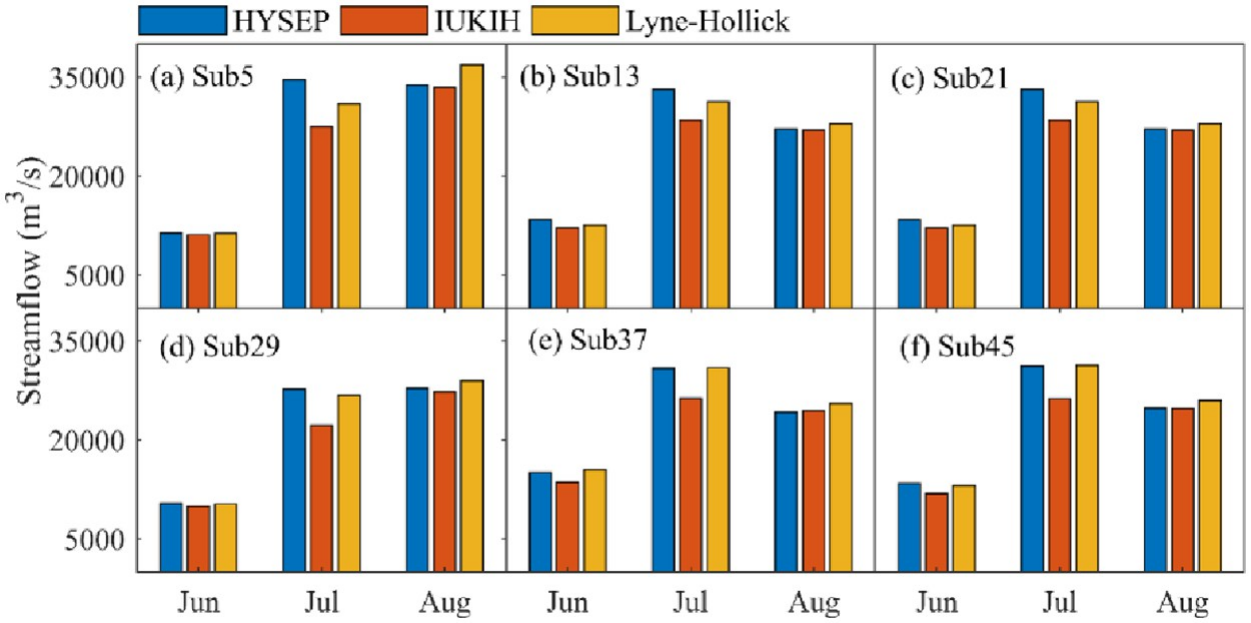
Influences of the base-flow segmentation method on water resources.

Based on the above discussion, the uncertainty influence of subbasin partitioning schemes and baseflow segmentation methods had a significant difference on baseflow processes in different periods. Taking June, July and August in 2008 as an example, Figs 12 and 13 represent the uncertainty influence of subbasin partitioning schemes and baseflow segmentation methods, respectively. The results show that the uncertainty influence of subbasin partitioning schemes was mainly reflected in the peak flow, and the lower the simulation accuracy was, the larger the peak flow value. In addition, the flow difference between the Sub37 scenario and Sub5 scenario on August 1, 2008, was as high as 680.23 m^3^/s.

Affected by factors, such as precipitation, temperature, model construction, etc., the baseflow segmentation methods had various manifestations, and the water resources varied significantly in different months. In addition, the larger the simulation accuracy was, the smaller the difference obtained by different baseflow segmentation methods. For example, in the Sub37 scenario, the monthly mean flow difference between the IUKIH and Lyne-Hollick methods in July was as high as 490.28 m^3^/s, but the difference between the HYSEP and Lyne-Hollick methods was small.

Therefore, the subbasin partitioning schemes and baseflow segmentation methods introduce great uncertainty to water resource assessments. The main task of this paper was to quantify the contribution of uncertainty of the subbasin partitioning schemes, the baseflow segmentation methods and their interaction effects in different periods to further evaluate the characteristics of water resource changes more accurately in the next stage.

## 6 Conclusions

The randomness and subjectivity of the selection of the subbasin partitioning schemes and baseflow segmentation methods resulted in significant differences in baseflow values in different months. Identifying and quantifying the uncertainty effects of the independence and interaction of multiple source factors can accurately assess the water resources in the basin. Therefore, a global sensitivity analysis framework is proposed to quantitatively assess the uncertainty influence of subbasin partitioning schemes and baseflow segmentation methods on baseflow processes in this paper. A number of important conclusions drawn from this study are as follows.

First, in the calibration and validation periods, the objective function values (*KGE*) in the Sub5, Sub13, Sub21, Sub29, Sub37 and Sub13 scenarios were 0.91 and 0.65, 0.94 and 0.86, 0.94 and 0.88, 0.92 and 0.82, 0.95 and 0.89, and 0.92 and 0.83, respectively. These findings indicate that subbasin partitioning scheme uncertainty has significant effects in the source region of the Yellow River.

Second, the baseflow varied with the change in simulated runoff, which was more obvious for the Lyne-Hollick and IUKIH methods, and the baseflow values estimated by the Lyne-Hollick and HYSEP methods were obviously higher than those estimated by the IUKIH method during the wet season.

Third, a global sensitivity analysis framework is proposed to quantitatively explore the uncertainty influence of subbasin partitioning schemes and baseflow segmentation methods on baseflow processes. The results show that the uncertainty influence of subbasin partitioning schemes was dominant in the dry season (January to March), accounting for 86%, the baseflow segmentation methods took second place, accounting for approximately 12%, and a combination of subbasin partitioning schemes and baseflow segmentation method uncertainty had the smallest impact, accounting for approximately 2%. From July to August, the uncertainty influence of the baseflow segmentation methods was gradually weakened, which may have been due to the uncertainty influence of the TOMODEL hydrological model. In the wet season (September to December), the uncertainty influence of subbasin partitioning schemes, baseflow segmentation methods and their interactions were significantly different, accounting for 50.63%, 48.33% and 1.04%, respectively.

In addition, although this study accurately identified the contributions of the subbasin division scheme and base flow segmentation method to the uncertainty of baseflow simulation in different periods, there were few hydrological models and base flow segmentation methods selected in this study, and the time series of the calibration model was short. More distributed hydrological models and base flow segmentation methods will be added in the future to verify the rationality of this study and to extend this research to more watersheds. Although the robustness of the research results needs to be further tested in other watersheds, the different uncertainty source assessment frameworks proposed in this study can also be applied to other watersheds.

## Supporting information

S1 Data Meteorological data. (XLSX)

S2 Data Hydrological data. (XLSX)

S3 Data Cover letter. (DOCX)
