# Supplementary material for: Quantifying interaction uncertainty between subwatersheds and base-flow partitions on hydrological processes
Source: PLoS One. 2022 Mar 1;17(3):e0261859. doi: 10.1371/journal.pone.0261859 (PMC8887730; doi:10.1371/journal.pone.0261859)
Supplement: S3 Data — (DOCX) [file pone.0261859.s003.docx]

Dear Editors:

Thank you very much for considering the publication of our manuscript in PLOS ONE. No conflict of interest exists in the submission of this manuscript, and the manuscript is approved by all authors for publication. I would like to declare on behalf of my co-authors that the work described was original research that has not been published previously, and has not been under consideration for publication elsewhere, in whole or in part. All the authors listed have approved the manuscript that is enclosed.

**Our response to the journal requirements is as follows:**

(1) We confirm that the author list and affiliations are correct on the title page of our manuscript, and that the author contributions, competing interests, and financial disclosure are correct. Below are the author's statement:

**Author Contributions:**

Bing Yan:

Conceptualization
Data curation
Resources
Writing – original draft

Yi Xu:

Writing – review & editing

**Competing Interests:** The authors have declared that no competing interests exist.

**Financial Disclosure:** This work was financially supported by the National Key Research and Development Program of China [Grant number: 2018YFC0407206, 2018YFC0406505] and the funders had no role in study design, data collection and analysis, decision to publish, or preparation of the manuscript.

(2) The meteorological data ((S1 Data File) in the manuscript can be downloaded at <https://1drv.ms/u/s!AuQ0zMqwfDDdkQ72Gjm06LdPRgk9?e=Hy32lj> publicly available to anyone. Meanwhile, we provide the contact address of the original data at http://data.cma.cn/data/cdcdetail/dataCode/A.0012.0001.html, which is available to everyone. But the account needs to be registered. Therefore, the account and password provided by us are as follows: **Login name:** [chxiang@xaut.edu.cn](mailto:chxiang@xaut.edu.cn); **Login password:** 2~c8KwqV!.

(3) We confirm that our paper meets PLOS ONE's typesetting requirements for References, Tables, and Figures.

(4) We have confirmed that each figure included in our submission files through the PACE tool and inserted the converted TIFF format picture into the manuscript.
